# Genetic prediction of lower limb isometric strength changes after 12 weeks of resistance training

**DOI:** 10.5114/biolsport.2025.145918

**Published:** 2025-01-14

**Authors:** Tao Mei, Yanchun Li, Dapeng Bao, Xiangang Yang, Xiaolin Yang, Liang Li, Zihong He

**Affiliations:** 1 China Institute of Sport and Health Science, Beijing Sport University, Beijing, China; 2 Beijing Key Laboratory of Sports Performance and Skill Assessment, Beijing Sport University, Beijing, China; 3 Hebei Sport University, Hebei, China; 4 Sultan Idris Education University, Tanjung Malin, Malaysia; 5 Biological Science Research Center, China Institute of Sport Science, Beijing, China

**Keywords:** Genome-wide association analysis, Isometric contraction, Resistance training, Individual differences, Predictive model

## Abstract

This study aimed to identify genetic variations associated with changes in isometric strength following resistance training and develop a predictive model for understanding training effects, providing insights for tailored fitness guidance. A 12-week resistance training program, consisting of traditional squats and bench press exercises, was completed by 193 healthy Chinese adults, with isometric strength assessed before and after the intervention. DNA was extracted for whole-genome genotyping, followed by genomewide association analysis using PLINK 1.9. Lasso regression was used to screen variables, and predictive models for training effectiveness were established using logistic regression, Nomogram, and stepwise linear regression. Following the training, participants showed an increased isometric strength (Δ = 8.69%, p = 0.08, ES = 0.07), ranging from -55.78% to 133.47%. Nineteen lead SNPs were significantly associated with improvements in lower limb isometric strength (p < 1 × 10^−5^), with 8 SNPs showing nominal associations (p < 1 × 10^−5^), and rs4623258 was the only SNP with genome-wide significance (p < 5 × 10^−8^). Stepwise linear regression identified several factors that impact training effects: baseline isometric strength, lower limb muscle mass, right rectus femoris length, rs4623258, rs344843, rs112298078, rs200507975, rs559077, rs8008364, rs6837485, rs4712860, rs76521421, rs1965365, and rs2746086 (adjusted R^2^ = 0.704). Logistic regression identified isometric strength, lean body mass, trunk fat, right rectus femoris length, rs139338397, rs76521421, rs8008364, and rs6579275 as significant factors influencing the outcome. (AUC = 0.875, p < 0.001). These findings show that predictive models can accurately predict changes in lower limb isometric strength after resistance training in Chinese subjects. However, applicability is primarily confined to East Asians, necessitating further studies in diverse populations to validate broader relevance.

## INTRODUCTION

Muscle mass loss and low muscle strength levels pose significant health challenges for sedentary individuals [1]. Lower limb strength is essential for maintaining normal daily activities and physical functions, impacting overall mortality rates, cardiovascular health, metabolic health, bone health, and quality of life [2]. Insufficient lower limb strength is closely associated with the risk of various chronic diseases and health issues, carrying substantial physiological and clinical significance [3, 4]. Isometric strength, an important indicator for assessing lower limb muscle strength, accurately reflects the maximal force muscles can exert in an isometric state. It is employed in clinical and scientific research as a crucial measure for monitoring health changes, diagnostics, rehabilitation, and evaluating the effectiveness of resistance training.

Resistance training is widely utilized to improve muscle strength, with its effectiveness in enhancing isometric strength supported by several studies [5, 6]. Meta-analyses have found that both moderate-to-high-load (> 60% 1RM) and lower-load (≤ 60% 1-RM) resistance training induces increases in isometric strength and muscle hypertrophy. Moreover, high-intensity training, younger participants, and untrained individuals tend to achieve more significant training effects [5]. Despite the potential of resistance training to enhance lower limb muscle strength, individual differences in improvements in isometric strength following resistance training exist among different subjects [7]. These differences are influenced by various factors such as individual physical fitness, and training experience, indicating variations in training effects among trained and untrained participants under different loads [8]. Additionally, individual differences are closely related to genetic factors, as human responsiveness to training is influenced by genetics. Research indicates that polymorphisms in peroxisome proliferator-activated receptors (*PPARs*) and their transcriptional coactivators are significantly associated with individual responses to specific aerobic training stimuli. Individuals carrying the *PPARGC1A* rs8192678 Gly/Gly, *PPARD* rs1053049 TT, *PPARD* rs2267668 AA, and *PPARG* rs1801282 Ala genotypes exhibit optimal responses to aerobic training, while those with the *PPARGC1A* rs8192678 Ser/Ser genotype show no response (e.g., no improvement in VO_2_peak, negligible increase in slow muscle fibers, and no significant decrease in low-density lipoprotein). Furthermore, individuals carrying the *PPARD* rs2267668 G allele may experience negative responses to aerobic training (e.g., a decrease in VO_2_peak) [9]. Muscle strength, encompassing isometric strength, dynamic strength, jumping ability, and other strength-related factors, exhibits a broad sense heritability estimate of approximately 0.51 [10]. Genetics similarly impact muscle strength concerning resistance training. Candidate gene studies have found some single nucleotide polymorphisms (SNPs) associated with the effectiveness of resistance training. For instance, *VEGFA* rs2010963 GG homozygotes exhibited higher maximal isometric strength increases in post-resistance training compared to carriers of the C allele [11]. *CCL2* rs1024610 T allele carriers experienced greater increases in maximal isometric strength post-resistance training [12]. Furthermore, *CNTF* rs1800169, *ACE* I/D, and *ACTN3* R577X have been reported to be associated with strength improvements after resistance training [13–15]. The effectiveness of training is a complex trait determined by multiple genes. Selecting training-effect-related SNPs at the whole-genome level is crucial for understanding individual differences, personalized training, and predicting training effects.

Genome-wide association analysis (GWAS) has played a significant role in identifying genetic variation points related to aerobic exercise capacity [16]. Regarding training effects, Vann et al. utilized a GWAS design and found that two SNPs (rs4675569 and rs10263647) in the *GLI3* gene were significantly associated with changes in muscle fiber cross-sectional area (fCSA). Participants with T/C and C/C genotypes exhibited more pronounced increases in fCSA, satellite cell numbers, and myonuclei following resistance training, whereas those with the T/T genotype did not show these changes [17]. These findings provide valuable insights for future research; however, there remains a paucity of GWAS-based studies on training effects, particularly in the context of resistance training.

Therefore, this study aims to conduct a GWAS using a Chinese resistance training cohort to identify genetic variations associated with individual differences in isometric strength changes post-resistance training. It aims to establish a predictive model for training effects using logistic regression, with the presence of training effects as the dependent variable, and to create a Nomogram plot. Additionally, it seeks to develop another predictive model using stepwise multiple regression, with the magnitude of training effects as the dependent variable. This research endeavors to provide robust scientific evidence for a better understanding of the mechanism of individual differences from a genetic perspective and for optimizing personalized fitness guidance programs.

## MATERIALS AND METHODS

### Participants

Inclusion criteria for participants were as follows: (1) Individuals classified as non-regular exercisers and having no history of resistance training during assessment using the Global Physical Activity Questionnaire (GPAQ). (2) Participants were determined to have no risk factors associated with resistance training based on the Physical Activity Readiness Questionnaire (PAR-Q). (3) Participants maintained regular dietary habits during the intervention period, as assessed by the Food Frequency Questionnaire from the Chinese Residents’ Nutrition and Health Survey, and did not consume dietary supplements that could affect muscle strength, such as protein powders (e.g., whey protein) or caffeine. All participants voluntarily joined the study and completed informed consent forms. A total of 193 Han Chinese participants were included in the study, comprising 95 males (mean age 20 ± 1 years, height 177.8 ± 5.8 cm, weight 71.3 ± 12.4 kg) and 98 females (mean age 20 ± 3 years, height 164.7 ± 5.9 cm, weight 56.5 ± 9.2 kg). Before the study, participants were briefed on the purpose and content of the research, and those who agreed to take part were selected. The study was conducted in accordance with the Declaration of Helsinki and approved by the Sports Science Experiment Ethics Committee (approval NO. 2019052H).

### Resistance Training Program

Prior to the resistance training intervention, participants performed a warm-up routine consisting of 5 minutes of jogging, followed by 5 minutes of dynamic stretching, and concluding with low-intensity resistance exercises. The objective of this warm-up was to elevate muscle temperature, enhance flexibility, and adequately prepare the participants for the subsequent resistance training session. Following this warm-up, subjects commenced the resistance training program. The regimen consisted of back squats and bench presses at 70% of their one-repetition maximum (1RM), totaling 5 sets of 10 repetitions each, with a 2-minute rest between sets, conducted twice a week for 12 weeks [18, 19]. Every 4 weeks, a 1RM test was administered to adjust the training load according to the changes in strength. Training loads for participants were monitored throughout the intervention to ensure adherence to proper exercise standards. Participants were encouraged to complete the full training volume. In cases where participants were unable to perform independently, minimal assistance was provided to ensure consistent training stimulus after completing the same training content. Indicator tests were conducted before and after the 12-week intervention period.

### Lower Limb Isometric Strength

The ISOMED 2000 dynamometer (ISOMED, Germany) was used to measure lower limb isometric strength [20]. Participants were positioned with the torso and thigh forming a 90° angle, and the lower leg at a 120° angle while fixed in place. Before the formal test, participants had three opportunities to practice bilateral leg pressing. After practice, they rested for 3 minutes before commencing the actual test. During testing, participants performed three maximal effort isometric leg presses with a 2-minute rest between each effort.

### One Repetition Maximum

Participants commenced with warm-up activities, including squatting/bench pressing at 40% of their perceived 1RM. Post-warm-up, the load was increased by 15–20 kilograms for 3–5 repetitions of squatting/bench pressing. Following a 2–4 minute rest, an additional 15–20 kilograms (for squatting) or 5–10 kilograms (for bench pressing) were added for 2–3 repetitions. Another rest interval of 2–4 minutes followed, repeating the previous steps. Weight was increased if the participant completed the lift; otherwise, it was reduced by 5–10 kilograms (for squatting) or 2.5–5 kilograms (for bench pressing) until achieving the correct 1RM. The determination for squatting and bench pressing 1RM was made within 5 attempts [21].

### Muscle Mass

The GE Lunar iDXA dual-energy X-ray absorptiometry system (GE Healthcare, USA) was utilized for measuring muscle mass. Before testing, participants ensured no barium meal inspections, radioactive isotope injections, or CT and MRI scans involving oral or injected contrast agents in the past 7 days. Participants fasted for at least two hours, removed any clothing that might interfere with test results, and lay flat on the scanner bed. Basic participant information was input into enCORE (2011), setting the scanning frame from head to foot for layer-by-layer scans to obtain muscle mass measurement data.

### Muscle Thickness

A color Doppler ultrasound machine (LOGIQ, USA) was used to measure the thickness of the rectus femoris, the distance between the rectus femoris and the vastus intermedius, and the pectoralis major. Muscle length was measured with a tape measure. (1) Rectus femoris marking: Participants lay flat with legs relaxed and shoulder-width apart, marking the midpoint between the anterior superior iliac spine and the upper edge of the patella. (2) Pectoralis major marking: Participants lay flat, using a body measuring tape. For males, the midpoint between the anterior axillary line and the nipple line was marked. For females, one-third of the distance from the anterior axillary line to the nipple was marked for measurement. (3) Rectus femoris to vastus intermedius marking: Using a 12 MHz linear array ultrasound probe, a B-mode scan obtained a crosssectional image perpendicular to the surface of the thigh at the midpoint. Each muscle was tested three times on each side, and the average was taken.

### Analysis of whole genome genetic polymorphisms

DNA extraction was performed using the Magnetic Bead Blood Genomic Extraction Kit (Tiangen Biotech). Genome-wide genotyping was conducted using the Infinium chip (CGA-24v1-0 type, Illumina). GenomeStudio 2.0 software was used to read the detection results and transform the data format.

Quality control methods for genotype imputation and imputation followed previous studies [22, 23]. Imputation was carried out using Eagle/Minimac4, with chunk size set at 10 MB and step size at 3 MB, utilizing the 1000 Genomes Project Phase 3 v5 haplotypes as reference. Quality control of the imputed chip data was performed using PLINK 1.9 software [24]: exclusions were based on (1) minor allele frequency < 5%; (2) deviations from Hardy-Weinberg equilibrium (p < 1 × 10^−5^); (3) SNPs with > 10% genotype missingness; and (4) individuals with > 10% genotype missingness. After genotype imputation, 4,110,727 SNPs were retained following quality control. Subsequent GWAS analysis was conducted using the quality-controlled SNPs.

### Data Analysis

Data entry, organization, and statistical analysis were performed using Excel 2016, SPSS 19.0, and R language packages. Descriptive statistics were presented as mean ± standard deviation (Mean ± SD). The normality of data distribution was assessed using the K-S test. Training effects were indicated by the percentage change in isometric strength before and after intervention (Δ). PCA was used for population stratification quality control. PLINK 1.9 software was employed for the GWAS analysis, incorporating initial isometric strength, gender, age, and the top 10 principal components from PCA as covariates, with significance set at p < 1 × 10^−5^ [17]. The genomic inflation factor (λ) was computed to assess bias in GWAS association results and the impact of population stratification. Manhattan and QQ plots for GWAS were generated using the “CMplot” package in R language.

Lasso regression was initially applied to the 116 variables (comprising 23 phenotypic variables and 93 genetic variants) for dimensionality reduction. Subsequently, logistic regression was employed to construct predictive models using the 93 genetic variants and 23 phenotypic indicators identified from GWAS as independent variables. In this analysis, categorical SNP data were meticulously encoded into dummy variables, with each SNP’s genotypes (AA, Aa, aa) represented separately to capture their individual effects on isometric strength outcomes. To simplify the model and mitigate multicollinearity, two dummy variables were typically included for each SNP, using the most common genotype as the reference group. Furthermore, nomogram plotting was utilized to visualize the relationships between the independent variables and the probability of isometric strength improvement post-intervention (categorized as Δ > 0 or Δ ≤ 0). Additionally, stepwise linear regression was conducted to identify the most significant predictors of the percentage change in isometric strength. This comprehensive approach enabled us to construct predictive models that accurately capture the influence of both genetic and phenotypic factors on isometric strength outcomes.

## RESULTS

**FIG. 1:**
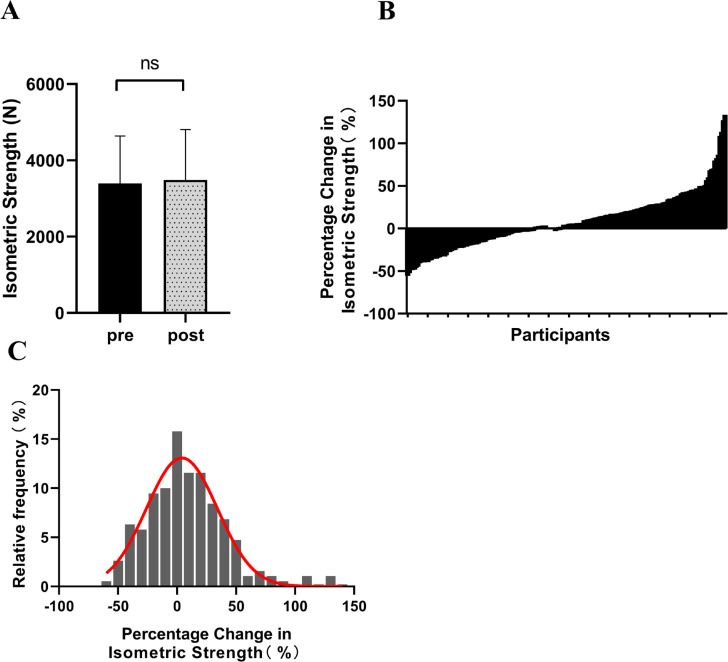
The changes in isometric strength among participants following resistance training (A: Isometric strength before and after intervention; B: Percentage change in participants’ isometric strength; C: Distribution of participants’ percentage change in isometric strength). ‘ns’ indicating p > 0.05 for the paired samples t-test.

After the 12-week intervention, there was an observable upward trend in isometric peak force, with the mean value increasing from 3391.51 ± 1247.95 N to 3489.54 ± 1316.80 N. However, this increase did not reach statistical significance (Δ = 8.69%, p = 0.08, ES = 0.07) (Figure 1A). Participants exhibited diverse changes in isometric strength, ranging from -55.78% to 133.47% (Figure 1B). Figure 1C depicts the distribution characteristics of isometric strength changes among participants, displaying a pattern largely consistent with a normal distribution (Figure 1C).

**FIG. 2:**
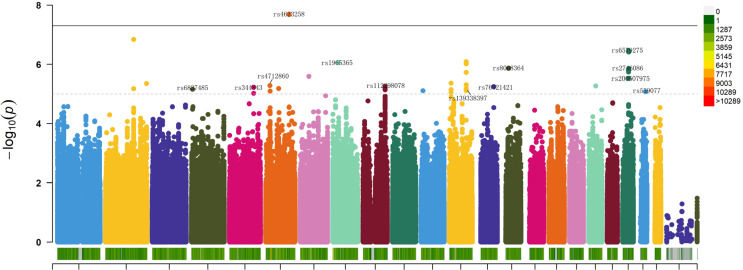
Manhattan plot depicting GWAS analysis of the impact of training on isometric leg press maximum strength. Chromosomes are represented on the x-axis, distinguished by various colors. The y-axis represents -log_10_ (P), with chromosome color in the legend indicating the quantity of SNPs on each chromosome. A dashed line indicates a significance level of p < 1×10^-5^, while a solid line represents a significance level of p < 5×10^-8^. SNPs included in the stepwise regression and logistic regression analyses are labeled.

The GWAS results revealed that 93 genetic variations were significantly associated with the enhancement of isometric strength in lower limbs (p < 1 × 10^−5^) (Figure 2), among which 19 were identified as lead SNPs (lead SNPs are typically the most significantly associated or strongly linked SNPs representing the genetic region, indicative of the variations in that genomic region) (Table 1). Rs4623258 was the only SNP with genome-wide significance (p < 5 × 10^−8^). The inflation factor λ = 1.019 indicated that the observed p-values are not a result of population stratification, indicating the absence of false positives.

**TABLE 1:** GWAS analysis of the training effect on isometric leg press maximum strength.

SNP ID	Chromosome	GRCh37 Position	REF Allele	Minor Allele	BETA	P	AFR freq	AMR freq	EUR freq	EAS freq	SAS freq
rs4623258	6	136063882	G	A	42.01	2.06E-08	0	0.07	0	0.04	0
rs74522496	2	164630637	A	G	43.2	1.45E-07	0	0	0	0.07	0
rs873925	20	34687450	T	A	34.29	3.46E-07	0.23	0.15	0.18	0.11	0.20
rs79663983	12	98376153	C	G	42.93	8.17E-07	0	0.03	0	0.05	0.03
rs1965365	8	31650080	A	A	39.16	8.70E-07	0.74	0.75	0.78	0.97	0.90
rs8008364	14	35366760	T	T	26.57	1.38E-06	0.62	0.88	0.9	0.89	0.83
rs11977136	7	49746444	G	A	22.32	2.55E-06	0.62	0.26	0.22	0.18	0.27
rs7135072	12	10345278	G	A	19.5	4.32E-06	0.29	0.52	0.54	0.22	0.42
rs10186582	2	239017580	C	A	18.49	4.42E-06	0.24	0.13	0.13	0.20	0.114
rs4712860	6	24837206	T	T	22.94	5.17E-06	0.39	0.59	0.47	0.86	0.56
rs4372771	18	40471759	C	T	25.11	5.35E-06	0.23	0.05	0.06	0.12	0.06
rs76521421	13	94671690	C	T	27.05	5.64E-06	0	0	0	0.04	0.001
rs344843	5	141406236	G	A	20.51	5.94E-06	0.07	0.17	0.26	0.20	0.13
rs7044663	9	128268263	C	T	16.92	6.06E-06	0.67	0.42	0.44	0.38	0.41
rs636444	6	75829467	G	T	21.71	6.55E-06	0.21	0.08	0.01	0.21	0.004
rs6837272	4	5808673	A	C	15.62	6.94E-06	0.32	0.4	0.46	0.37	0.47
rs10831636	11	11558051	A	A	19.08	7.85E-06	0.62	0.5	0.39	0.80	0.43
rs78983140	6	25381967	A	G	-18.11	8.07E-06	0.01	0.06	0.04	0.28	0.11
rs559077	21	41028375	C	T	30.76	8.40E-06	0.26	0.33	0.48	0.06	0.45

**FIG. 3:**
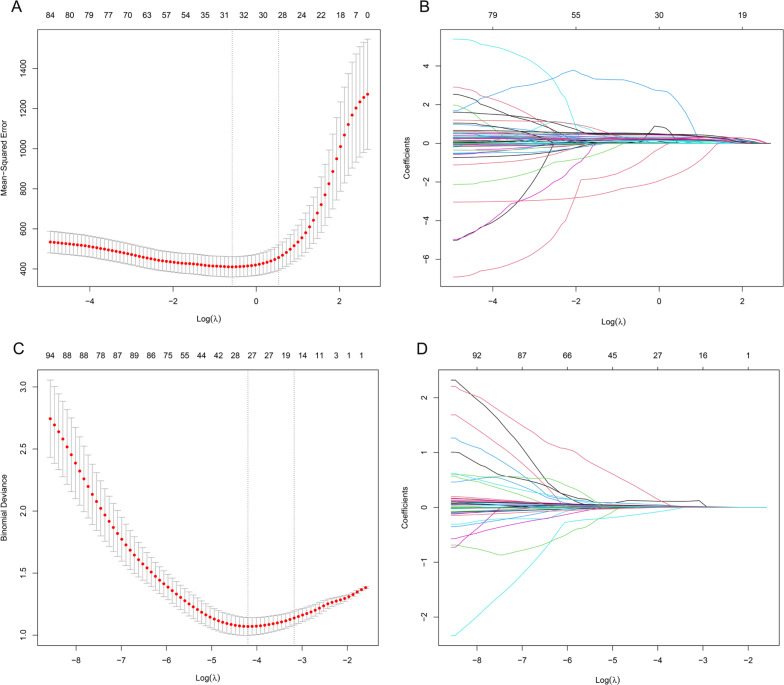
Lasso Regression Cross-Validation Curve and Coefficient Path Plot. A and B perform variable selection using Lasso regression prior to stepwise regression, while C and D perform variable selection using Lasso regression prior to logistic regression. The x-axis of the A and C represents log(λ), while the y-axis represents mean error. The optimal λ corresponds to the minimum average error. B and D illustrates how coefficients are compressed with changing log(λ). Variables where the coefficients are non-zero at the optimal λ on the y-axis are selected as independent variables for subsequent modeling.

Lasso regression was used for dimensionality reduction on 116 variables (23 phenotypic indicators and 93 genetic variant loci) to select the most representative influencing factors. A 10-fold cross-validation determined the optimal Lambda (λ) parameter, selecting λ = 0.56 to minimize cross-validation error (Figure 3A, 3B). The results indicated that 8 phenotypic indicators (gender, baseline isometric strength, lean body mass, overall body fat, lower limb muscle mass, right quadriceps length, right quadriceps and thigh muscle thickness, left pectoral muscle thickness) and 27 SNPs (including rs74522496, rs111655501, rs4372771, rs7875713, rs200317032, rs7135072, rs78983140, rs2746086, rs11977136, rs1965365, rs112298078, rs10831636, rs6837272, rs559077, rs4623258, rs4712860, rs8008364, rs76521421, rs344843, rs6141595, rs10186582, rs6837485, rs164512, rs2223439, rs636444, rs7044663, rs7865246), totaling 35 variables, influence the efficacy of changes in isometric strength following resistance training.

Isometric strength change post-intervention was used as the dependent variable, with 35 variables selected by Lasso regression as independent variables for stepwise linear regression. Baseline isometric strength, lower limb muscle mass, right quadriceps length, and SNPs (rs4623258, rs344843, rs112298078, rs200507975, rs559077, rs8008364, rs6837485, rs4712860, rs76521421, rs1965365, rs2746086) significantly impacted training effect (R^2^ = 0.727, adjusted R^2^ = 0.704, Table 2).

**TABLE 2:** Results of Stepwise Linear Regression Analysis

Coefficient	Unstandardized Coefficients		Standardized Coefficients	P-value	R^2^	Adjusted R^2^

	B	Sth.Erro	BETA			
Constant	52.537	27.032		0.054		
Baseline isometric strength	-0.014	0.002	-0.506	< 0.001	0.169	0.164
rs4623258	0.409	0.118	0.148	0.001	0.132	0.129
rs344843	0.62	0.137	0.189	< 0.001	0.077	0.075
rs112298078	0.452	0.136	0.139	0.001	0.066	0.064
rs200507975	0.418	0.166	0.128	0.013	0.058	0.057
rs559077	0.731	0.141	0.214	< 0.001	0.057	0.056
rs8008364	0.604	0.131	0.19	< 0.001	0.045	0.044
rs6837485	0.585	0.139	0.177	< 0.001	0.029	0.028
Lower limb muscle mass	0.003	0.001	0.327	< 0.001	0.031	0.03
rs4712860	0.453	0.137	0.14	0.001	0.016	0.015
rs76521421	0.406	0.143	0.121	0.005	0.016	0.015
Right rectus femoris muscle length	-1.789	0.723	-0.137	0.014	0.013	0.012
rs1965365	0.306	0.136	0.098	0.026	0.01	0.009
rs2746086	0.342	0.153	0.111	0.027	0.008	0.006

Dimensionality reduction from 116 variables was conducted using Lasso regression to isolate key influencing factors. The optimal penalty coefficient Lambda (λ) was selected via 10-fold cross-validation, achieving the lowest cross-validation error (Figure 3C, 3D). λ = 0.015 was determined as optimal, considering non-zero regression coefficients. These variables, representing key influencers, were analyzed further. Phenotypic indicators (gender, baseline isometric strength, lean body mass, trunk fat mass, right rectus femoris length, left pectoralis major thickness) and 29 SNPs (including rs78983140, rs2223439, rs873925, rs200507975, rs7865246, rs67619039, rs6837485, rs74522496, rs79417728, rs7875713, rs10186582, rs7135072, rs11977136, rs2746091, rs6579275, rs8008364, rs112298078, rs4372771, rs111655501, rs76521421, rs4712860, rs6141595, rs10831636, rs6837272, rs4623258, rs636444, rs344843, rs1965365, rs559077) collectively formed a total of 35 variables that significantly influenced the effectiveness of changes in isometric strength following resistance training.

**FIG. 4:**
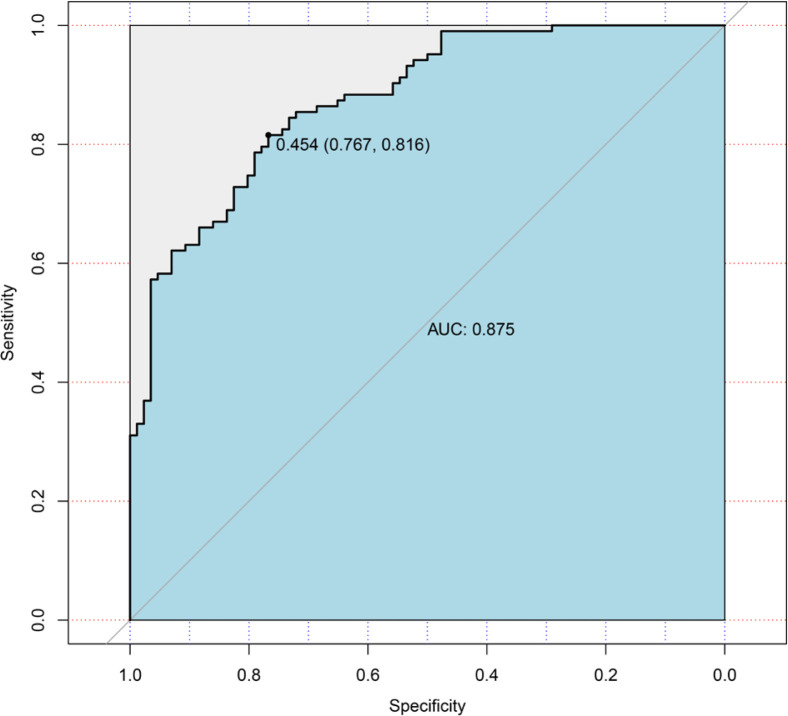
The area under the curve (AUC).

**FIG. 5:**
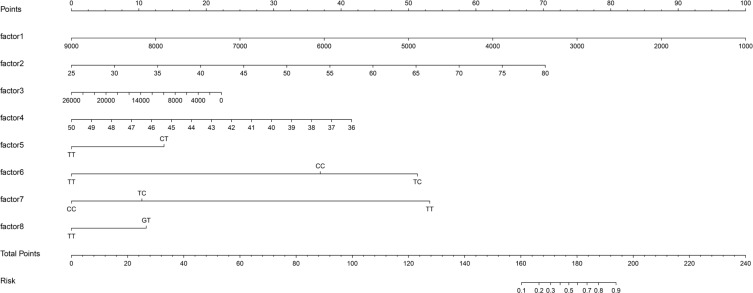
Nomogram Predictive Model Line Chart. factor1: isometric leg strength; factor2: lean body mass; factor3: trunk fat; factor4: right quadriceps length; factor5: rs139338397; factor6: rs76521421; factor7: rs8008364; factor8: rs6579275. When the information about factors 1-8 of the subjects is known, draw a vertical line on the axis to obtain the Points corresponding to each factor. Summing these Points gives the Total Points, determining the associated risk probability Risk.

Logistic regression used the improvement status of isometric leg strength post-intervention (Δ > 0 or Δ ≤ 0) as the dependent variable, with the same 35 Lasso-screened variables as independent variables. Isometric leg strength, lean body mass, trunk fat, right quadriceps length, and SNPs (rs139338397, rs76521421, rs8008364, rs6579275) significantly influenced training effectiveness (Table 3). The ROC curve indicated an AUC of 0.875 (p < 0.001), with a threshold of 0.454 (Figure 4). The Nomogram illustrated that a total score below 160 indicated ineffective training for enhancing isometric leg strength (Figure 5).

**TABLE 3:** Results of Logistic Regression Analysis

Coefficient	B	S.E,	Wals	P-value	Exp (B)
Constant	19.753	56840.329	1.208E-7	1.000	3.79E+08
Baseline isometric strength	-0.002	2.867E-4	32.383	< 0.001	0.998
Lean body mass	0.164	0.036	20.523	< 0.001	1.178
Trunk fat mass	-1.063E-4	4.979E-5	4.562	0.033	1
Right rectus femoris muscle length	-0.381	0.111	11.724	0.001	0.683
rs139338397(1)	-1.820	0.948	3.687	0.055	0.162
rs76521421			7.13	0.028	
rs76521421(1)	18.746	40192.654	2.175E-7	1.000	1.38E+08
rs76521421(2)	20.64	40192.654	2.637E-7	1.000	9.20E+08
rs8008364			6.573	0.037	
rs8008364(1)	-20.93	40192.652	2.712E-7	1.000	8.12E-10
rs8008364(2)	-19.537	40192.652	2.363E-7	1.000	3.28E-9
rs6579275(1)	-1.468	0.748	3.858	0.050	0.230

## DISCUSSION

This study primarily delineates individual variations in the enhancement of isometric leg strength following resistance training. From a genomic perspective, we identified 93 genetic variations associated with the improvement of isometric leg strength. The established predictive model for training effectiveness showed promising predictive performance for the enhancement of isometric leg strength following resistance training (AUC = 0.875). Based on this, we introduced a Nomogram prediction model for the first time, specifically for forecasting improvements in isometric leg strength after resistance training. According to this model, a total score below 160 indicates that the training is unlikely to have a meaningful impact on enhancing isometric leg strength. Furthermore, the stepwise linear regression model effectively predicted the degree of improvement in isometric strength following resistance training.

Resistance training is generally believed to be beneficial for enhancing isometric muscle strength, as consistently proven in most studies. A 13-week dynamic resistance training with intensities ranging from 30% to 60% 1 RM significantly increased isometric strength by 4.8% [25]. Another study conducted for 21 weeks with resistance training intensities from 50% to 80% 1 RM showed a 22% increase in 1RM load and a 21% increase in maximum isometric strength in both resistance training alone and combined strength and endurance training conditions [26]. Meta-analyses have also shown that both high and low-load resistance training can significantly improve isometric muscle strength, averaging a 21.5 ± 5.3% increase (95% CI: 8.9–34.2) [27], similar to the results of this study. Despite ample evidence demonstrating the effectiveness of such training in enhancing muscle strength, its effects may vary among individuals, demonstrating individual differences.

Several studies have reported individual differences in strength improvement during resistance training. For instance, a study revealed individual differences in strength improvement after a 9-week leg progressive resistance training, where participants exhibited varying outcomes across multiple metrics: MVC torque increased by an average of 26% ± 11%, ranging from -1% to 52%, quadriceps maximum muscle strength increased by an average of 22% ± 11%, ranging from -1% to 44%, and physiological cross-sectional area increased by an average of 6 ± 4%, ranging from -3% to 18% [28]. These studies indicate that resistance training does not universally lead to improved muscle strength when individual differences are taken into account; in fact, some participants experienced a decline in muscle strength. Regarding isometric strength, a study after a 12-week full-body resistance training demonstrated individual differences in the improvement of isometric leg strength, where about one-third of participants experienced a decrease or no change in isometric and isokinetic torque, while another third showed moderate increases, and the remaining third exhibited significant increases [7], resembling the results of this study.

Individual differences in training effects might be attributed to various factors such as genetic elements, physiological disparities, training levels, nutrition, and lifestyle. Genomic variants (e.g., genetic variations in *CNTF, ACTN3, IGF2, MSTN*, and *VDR* genes) may impact muscle growth, protein synthesis rates, and neural regulation, among other aspects [29], resulting in varying individual responses to training. Physiological differences like hormones, muscle fiber types, and neural control are also influential factors affecting strength. From a training perspective, the experience level and training history in resistance training may influence an individual’s response to training. Individuals with less or no prior exposure to similar training might experience more significant enhancements, whereas experienced trainees might have less room for improvement.

This study endeavors to elucidate individual differences in improved isometric lower limb strength after resistance training at the genomic level. Earlier researchers used a candidate gene approach and discovered several genetic variation loci associated with resistance training. Gene polymorphisms like *ACE, ACTN3, IGF1, IGFBP3, PPP3R1, CNTF, MSTN, B2BRK, PTK2, CCL2, CCR2, IL15*, and *IL-15RA* have been extensively reported as genetic variation loci related to resistance training effects [16]. For example, in a 12-week unilateral arm resistance training, the *ACE* I/D genotype explained approximately 1% of MVC in the trained arm and about 2% of MVC, 2% of 1RM, and 4% of CSA in the untrained arm in response to resistance training [14]. However, conflicting study results exist; *ACE* I/D polymorphism was associated with baseline muscle volume in females (aged 50–85 years) but showed no association with baseline strength or resistance training response [30]. Inconsistencies in study outcomes might be due to the limited explanatory power of individual genetic variation loci on training effects, where other phenotype factors (such as physiological differences and training levels) overshadow the role of genetic variation, rendering non-genetic variation irrelevant to training response. This necessitates the use of GWAS to screen genetic variation loci and explore the role of multiple genes in resistance training response.

GWAS has been utilized to discover genetic variations associated with strength phenotypes. In a GWAS study involving 195,180 participants, 16 loci associated with grip strength (P < 5 × 10^−8^) were identified, some of which contained genes related to skeletal muscle fiber structure and function (e.g., *ACTG1*), nerve maintenance and signal transduction (e.g., *PEX14, TGFA, SYT1*), or single-gene syndromes related to psychiatric motor disorders (e.g., *PEX14, LRPPRC*, and *KANSL1*) [31]. These findings provided new insights into genetic determinants explaining individual differences in grip strength while also highlighting the advantages of GWAS in identifying new genetic variations. However, studies explaining training effects from a genomic level using large tissue samples are immensely challenging.

This study presents the first GWAS results of improved isometric lower limb strength following resistance training, identifying 93 SNPs associated with isometric strength improvement. In selecting the screening threshold for SNPs, we set it at P < 1 × 10^−5^. This decision was primarily based on the relatively small sample size of our study. In smaller sample sizes, employing a more stringent p-value cutoff (such as 1 × 10^−8^) can lead to a substantial number of false negatives, resulting in missed detection of actual associations. Therefore, to balance the risks of false positives and false negatives, we opted for a more lenient p-value threshold. This approach has also been utilized in previous studies to maximize the capture of potential association signals within limited sample sizes [17, 32].

To further validate the functional relevance of the identified 19 lead SNPs, we analyzed available datasets from the GTEx portal (Genotype-Tissue Expression project) [33]. Among the 19 lead SNPs, rs873925, rs8008364, rs7135072, rs10186582, rs4712860, rs4372771, rs344843, rs7044663, rs6837272, rs78983140, and rs559077 serve as eQTLs (expression quantitative trait loci) and play pivotal roles in regulating gene expression across various tissues. Specifically, rs873925, rs10186582, rs7044663, and rs78983140 are identified as eQTLs for skeletal muscle, with normalized effect sizes (NES) of -0.21, 0.52, -0.12, and -0.25, respectively. Similarly, rs873925, rs8008364, rs7135072, rs10186582, rs4712860, and rs7044663 function as eQTLs for the testis, exhibiting NES values of -0.49, -0.26, 0.24, 0.72, -0.26, and 0.17, respectively. However, significant eQTLs were not found for rs4623258, rs74522496, rs79663983, rs1965365, rs11977136, rs76521421, rs636444, and rs10831636 across all examined tissues, although these SNPs may still play important roles in other biological processes.

Among the selected SNPs, rs1965365 is situated in the Neuregulin 1 (*NRG1*) gene. *NRG1* is a growth factor produced by peripheral nerves and skeletal muscles. In muscles, it regulates gene expression related to neuromuscular junctions, acetylcholine receptor numbers, muscle homeostasis, and satellite cell survival. Muscle spindles, specialized receptors in muscle tissue sensing and regulating muscle tension and length, are crucially maintained by Nrg1, essential for motor coordination [34]. Nrg1 plays a role in muscle injury and resists muscle atrophy [35]. rs4712860 is located on the *FAM65B* gene, and current evidence suggests that Fam65b might play a significant role in skeletal muscle development and function, with reduced Fam65b expression resulting in abnormalities and lethality in zebrafish muscle tissue [36]. Thirteen SNPs (e.g., rs7044663, rs12351538, rs10663901) are located on the *MAPKAP1* gene, typically associated with the MAP kinase signaling pathway. While its precise role in skeletal muscles may vary across studies and conditions, research has indicated that MAPKAP1/MK2 might play a regulatory role in muscle inflammation, muscle development, and exercise adaptability [37]. These regulatory relationships might provide clues about how SNPs influence the improvement of isometric strength following resistance training.

Furthermore, some SNPs are situated among non-coding RNAs; for instance, rs4623258, the most significant SNP, lies between *LINC00271* and *RP11-394G3.2* lincRNAs. While lincRNAs do not encode proteins, they play crucial roles in gene expression regulation, cell cycle control, cell differentiation, signal transduction, and disease occurrence. The limited research associating *LINC00271* and *RP11-394G3.2* with skeletal muscle phenotypes requires further validation. Presently, there is limited widespread research reporting the relationship and roles of other SNPs’ host genes like *GALNT18, LRRC16A*, and *EPB41L1* with skeletal muscles. Future studies may need to further explore these SNPs and their host genes’ potential links to skeletal muscle health, metabolism, and functionality.

Training effects are determined by a combination of factors like genetics, phenotype, and environment, among others, making them somewhat predictable. For instance, the percentage of type II muscle fibers and the cross-sectional area of type II muscle fibers can predict 15.2% and 20.7% of muscle hypertrophy score changes after a six-week high-intensity resistance training [38]. Studies found that matching high or low-intensity resistance training programs with an individual’s genotype more effectively enhances athletes’ explosiveness (CMJ) and aerobic 3-minute cycle test, indicating that aligning individual genotypes with appropriate training modalities can optimize resistance training [39]. In this study, the model incorporating phenotype and genetic variation factors exhibited an overall explanatory capacity of 70.4% (sum of adjusted R^2^), effectively predicting improvements in isometric lower limb strength post-resistance training. Among phenotype factors, baseline isometric strength, lower limb muscle mass, and right rectus femoris length were predictors of improvements in isometric lower limb strength, explaining 20.6% of the strength increase. After a 12-week isometric knee extension resistance training, baseline MVT values could account for 10.6% of ΔMVT changes, and quadriceps muscle volume at baseline could explain 18.7% of ΔMVT changes. MVT baseline values exhibited a negative correlation with ΔMVT, while quadriceps muscle volume baseline values showed a positive correlation [40], consistent with trends observed in this study. This study reported that rectus femoris length is one of the influencing factors in changes to isometric lower limb strength post-resistance training, a rare report on its impact on resistance training effects. However, resistance training can influence the anatomical structure of the rectus femoris, such as cross-sectional area and thickness [41].

It’s widely acknowledged that genetic variation possibly accounts for a significant portion of heterogeneity in training response. For example, a meta-analysis found that 72% of the total variability in 1RM strength gains post-training intervention could be explained through genetic grouping, involving genes like *ACE, ACTN3, AKT1, COX4I1, MTOR*, and *VEGFA* [42]. Regarding genetic factors, this study found that 11 SNPs including rs4623258, rs344843, rs112298078, rs200507975, rs559077, rs8008364, rs6837485, rs4712860, rs76521421, rs1965365, and rs2746086 collectively explained 49.8% of isometric strength gains. This indicates a more significant role of genetic variation in determining improvements in isometric lower limb strength post-resistance training. Among these, rs4623258 stands out as the most significant SNP, while rs1965365 and rs4712860 are located on *NRG1* and *FAM65B* genes, respectively, both closely linked to skeletal muscle growth and physiological processes.

Logistic regression was employed to predict training effects (effectiveness), incorporating several phenotype indicators (baseline isometric strength, body fat weight, trunk fat, and right rectus femoris length) and genetic variations (rs139338397, rs76521421, rs8008364, rs6579275) into the model. Baseline isometric strength, trunk fat, and right rectus femoris length, along with rs139338397, rs8008364, and rs6579275, exhibited odds ratios (OR) less than 1, indicating a negative correlation with training effects. Conversely, body fat weight and rs76521421 showed OR values greater than 1, indicating a positive correlation. Model performance was assessed using AUC (area under the curve), yielding a value of 0.875, indicating high predictive capability in identifying responders to resistance training. This study introduced a Nomogram for the first time to predict training effects, visually displaying logistic regression results, where a total score < 160 suggests ineffective improvement in isometric strength. Research on predicting responders to resistance training remains limited, making this study’s approach novel and valuable.

In this study, we investigated the differences in allele frequencies across various populations. Notably, our data revealed the absence of certain minor alleles (such as rs76521421 and rs74522496) in certain non-Asian populations. This observation holds significant importance for understanding the distribution of genetic variations across different racial and geographical groups. Due to the absence of these minor alleles in non-Asian populations, our model may encounter limitations when predicting and interpreting the genetic characteristics of these populations. Therefore, we clarify here that the model constructed in this study is primarily based on genetic data from Chinese individuals and is applicable to the Chinese population. For non-Asian populations, the applicability of the model requires further validation and adjustment. We anticipate that future research will extend to more diverse populations to further validate and optimize our model, thereby providing a more comprehensive understanding of the impact of genetic variations on isometric muscle strength.

## LIMITATIONS

This study has several limitations regarding the establishment of predictive models. First, although the research focused on phenotypic and genetic factors, it may not account for all possible influencing factors, such as nutrition, lifestyle habits, psychological states, and training history. These factors can significantly impact the effectiveness of resistance training; therefore, future studies should explore these potential predictors to enhance the comprehensiveness and accuracy of the models. Second, the design of the resistance training protocol in this study did not take into consideration the influence of training tempo. Training tempo, defined as the speed and timing of each exercise movement, can significantly affect physiological responses and outcomes [43]. Variations in tempo may lead to differences in muscle stimulation and adaptation, potentially impacting the predictive accuracy of the models. Future research should incorporate training tempo as a variable to assess its potential effects on training outcomes. Lastly, the subjects of this study were exclusively Asian individuals, which may limit the generalizability of the findings to other populations, such as Europeans and Africans. Future research should aim to validate these predictive models across diverse ethnic groups to ensure their broader applicability and effectiveness.

## CONCLUSIONS

In this study, we identified ninety-three genetic variations that were associated with improvements in lower limb isometric strength following resistance training. Notably, rs4623258 reached genome-wide significance, indicating its potential importance in this context. Utilizing these genetic variations and phenotypic indicators, we developed Logistic predictive models, Nomogram plots, and stepwise regression models, which demonstrated effective predictive capability for changes in lower limb isometric strength specifically among Chinese subjects. The Nomogram plots provided a practical tool for assessing the likelihood of training-induced improvements in isometric strength, with a total score below 160 suggesting a limited effect. However, it is important to acknowledge that the applicability of our model is currently confined primarily to East Asians. Therefore, further replication studies in diverse populations are necessary to validate the broader relevance of our findings. Such studies will help to establish the generalizability of our predictive models and contribute to a more comprehensive understanding of the genetic determinants of training-induced improvements in isometric strength.

## References

[1] PiPinto AJ, Bergouignan A, Dempsey PC, Roschel H, Owen N, Gualano B, Dunstan DW. Physiology of sedentary behavior. Physiol Rev. 2023;103(4):2561–2622. 37326297 10.1152/physrev.00022.2022 PMC10625842

[2] Ramari C, Hvid LG, David AC, Dalgas U. The importance of lower-extremity muscle strength for lower-limb functional capacity in multiple sclerosis: Systematic review. Ann Phys Rehabil Med. 2020;63(2):123–137. 31816449 10.1016/j.rehab.2019.11.005

[3] Lopez-Jaramillo P, Lopez-Lopez JP, Tole MC, Cohen DD. Muscular Strength in Risk Factors for Cardiovascular Disease and Mortality: A Narrative Review. Anatol J Cardiol. 2022;26(8):598–607. 35924286 10.5152/AnatolJCardiol.2022.1586 PMC9403882

[4] Yildiz S, Glanzman AM, Estilow T, Flickinger J, Brandsema JF, Tennekoon G, Banwell BL, Yum S. Retrospective Analysis of Fractures and Factors Causing Ambulation Loss After Lower Limb Fractures in Duchenne Muscular Dystrophy. Am J Phys Med Rehabil. 2020;99(9):789–794. 32195737 10.1097/PHM.0000000000001423

[5] Refalo MC, Hamilton DL, Paval DR, Gallagher IJ, Feros SA, Fyfe JJ. Influence of resistance training load on measures of skeletal muscle hypertrophy and improvements in maximal strength and neuromuscular task performance: A systematic review and meta-analysis. J Sports Sci. 2021;39(15):1723–1745. 33874848 10.1080/02640414.2021.1898094

[6] Ogawa M, Hashimoto Y, Mochizuki Y, Inoguchi T, Kouzuma A, Deguchi M, Saito M, Homma H, Kikuchi N, Okamoto T. Effects of free weight and body mass-based resistance training on thigh muscle size, strength and intramuscular fat in healthy young and middle-aged individuals. Exp Physiol. 2023;108(7):975–985. 37133323 10.1113/EP090655 PMC10988481

[7] Clark LA, Russ DW, Tavoian D, Arnold WD, Law TD, France CR, Clark BC. Heterogeneity of the strength response to progressive resistance exercise training in older adults: Contributions of muscle contractility. Exp Gerontol. 2021;152:111437. 34098008 10.1016/j.exger.2021.111437 PMC8319076

[8] Lacio M, Vieira JG, Trybulski R, Campos Y, Santana D, Filho JE, Novaes J, Vianna J, Wilk M. Effects of Resistance Training Performed with Different Loads in Untrained and Trained Male Adult Individuals on Maximal Strength and Muscle Hypertrophy: A Systematic Review. Int J Environ Res Public Health. 2021;18(21): 11237. 34769755 10.3390/ijerph182111237 PMC8582674

[9] Petr M, Stastny P, Zajac A, Tufano JJ, Maciejewska-Skrendo A. The Role of Peroxisome Proliferator-Activated Receptors and Their Transcriptional Coactivators Gene Variations in Human Trainability: A Systematic Review. Int J Mol Sci. 2018;19(5): 1472. 29762540 10.3390/ijms19051472 PMC5983571

[10] Zempo H, Miyamoto-Mikami E, Kikuchi N, Fuku N, Miyachi M, Murakami H. Heritability estimates of muscle strength-related phenotypes: A systematic review and meta-analysis. Scand J Med Sci Sports. 2017;27(12):1537–1546. 27882617 10.1111/sms.12804

[11] Boidin M, Dawson EA, Thijssen DHJ, Erskine RM. VEGFA rs2010963 GG genotype is associated with superior adaptations to resistance versus endurance training in the same group of healthy, young men. Mol Genet Genomics. 2023;298(1):119–129. 36326960 10.1007/s00438-022-01965-4 PMC9816297

[12] Harmon BT, Orkunoglu-Suer EF, Adham K, Larkin JS, Gordish-Dressman H, Clarkson PM, Thompson PD, Angelopoulos TJ, Gordon PM, Moyna NM, Pescatello LS, Visich PS, Zoeller RF, Hubal MJ, Tosi LL, Hoffman EP, Devaney JM. CCL2 and CCR2 variants are associated with skeletal muscle strength and change in strength with resistance training. J Appl Physiol (1985). 2010;109(6):1779–1785. 20947712 10.1152/japplphysiol.00633.2010 PMC3006412

[13] Walsh S, Kelsey BK, Angelopoulos TJ, Clarkson PM, Gordon PM, Moyna NM, Visich PS, Zoeller RF, Seip RL, Bilbie S, Thompson PD, Hoffman EP, Price TB, Devaney JM, Pescatello LS. CNTF 1357 G -> A polymorphism and the muscle strength response to resistance training. J Appl Physiol (1985). 2009;107(4):1235–1240. 19628720 10.1152/japplphysiol.90835.2008 PMC2763829

[14] Pescatello LS, Kostek MA, Gordish-Dressman H, Thompson PD, Seip RL, Price TB, Angelopoulos TJ, Clarkson PM, Gordon PM, Moyna NM, Visich PS, Zoeller RF, Devaney JM, Hoffman EP. ACE ID genotype and the muscle strength and size response to unilateral resistance training. Med Sci Sports Exerc. 2006;38(6):1074–1081. 16775548 10.1249/01.mss.0000222835.28273.80

[15] Clarkson PM, Devaney JM, Gordish-Dressman H, Thompson PD, Hubal MJ, Urso M, Price TB, Angelopoulos TJ, Gordon PM, Moyna NM, Pescatello LS, Visich PS, Zoeller RF, Seip RL, Hoffman EP. ACTN3 genotype is associated with increases in muscle strength in response to resistance training in women. J Appl Physiol (1985). 2005;99(1):154–163. 15718405 10.1152/japplphysiol.01139.2004

[16] Ahmetov, II, Hall ECR, Semenova EA, Pranckeviciene E, Gineviciene V. Advances in sports genomics. Adv Clin Chem. 2022;107:215–263. 35337603 10.1016/bs.acc.2021.07.004

[17] Vann CG, Morton RW, Mobley CB, Vechetti IJ, Ferguson BK, Haun CT, Osburn SC, Sexton CL, Fox CD, Romero MA, Roberson PA, Oikawa SY, McGlory C, Young KC, McCarthy JJ, Phillips SM, Roberts MD. An intron variant of the GLI family zinc finger 3 (GLI3) gene differentiates resistance training-induced muscle fiber hypertrophy in younger men. FASEB J. 2021;35(5):e21587. 33891350 10.1096/fj.202100113RR PMC8234740

[18] Lopez P, Radaelli R, Taaffe DR, Newton RU, Galvao DA, Trajano GS, Teodoro JL, Kraemer WJ, Hakkinen K, Pinto RS. Resistance Training Load Effects on Muscle Hypertrophy and Strength Gain: Systematic Review and Network Meta-analysis. Med Sci Sports Exerc. 2021;53(6):1206–1216. 33433148 10.1249/MSS.0000000000002585 PMC8126497

[19] Schoenfeld BJ, Ogborn D, Krieger JW. Effects of Resistance Training Frequency on Measures of Muscle Hypertrophy: A Systematic Review and Meta-Analysis. Sports Med. 2016;46(11):1689–1697. 27102172 10.1007/s40279-016-0543-8

[20] Dirnberger J, Wiesinger H-P, Kösters A, Müller E. Reproducibility for isometric and isokinetic maximum knee flexion and extension measurements using the IsoMed 2000-dynamometer. Isokinet Exerc Sci. 2012;20:149–153.

[21] Seo DI, Kim E, Fahs CA, Rossow L, Young K, Ferguson SL, Thiebaud R, Sherk VD, Loenneke JP, Kim D, Lee MK, Choi KH, Bemben DA, Bemben MG, So WY. Reliability of the one-repetition maximum test based on muscle group and gender. J Sports Sci Med. 2012;11(2):221–225. 24149193 PMC3737872

[22] Anderson CA, Pettersson FH, Clarke GM, Cardon LR, Morris AP, Zondervan KT. Data quality control in genetic case-control association studies. Nat Protoc. 2010;5(9):1564–1573. 21085122 10.1038/nprot.2010.116 PMC3025522

[23] van Leeuwen EM, Kanterakis A, Deelen P, Kattenberg MV, Genome of the Netherlands C, Slagboom PE, de Bakker PI, Wijmenga C, Swertz MA, Boomsma DI, van Duijn CM, Karssen LC, Hottenga JJ. Population-specific genotype imputations using minimac or IMPUTE2. Nat Protoc. 2015;10(9):1285–1296. 26226460 10.1038/nprot.2015.077

[24] Marees AT, de Kluiver H, Stringer S, Vorspan F, Curis E, Marie-Claire C, Derks EM. A tutorial on conducting genomewide association studies: Quality control and statistical analysis. Int J Methods Psychiatr Res. 2018;27(2):e1608. 29484742 10.1002/mpr.1608 PMC6001694

[25] Alegre LM, Jimenez F, Gonzalo-Orden JM, Martin-Acero R, Aguado X. Effects of dynamic resistance training on fascicle length and isometric strength. J Sports Sci. 2006;24(5):501–508. 16608764 10.1080/02640410500189322

[26] Hakkinen K, Alen M, Kraemer WJ, Gorostiaga E, Izquierdo M, Rusko H, Mikkola J, Hakkinen A, Valkeinen H, Kaarakainen E, Romu S, Erola V, Ahtiainen J, Paavolainen L. Neuromuscular adaptations during concurrent strength and endurance training versus strength training. Eur J Appl Physiol. 2003;89(1):42–52. 12627304 10.1007/s00421-002-0751-9

[27] Schoenfeld BJ, Grgic J, Ogborn D, Krieger JW. Strength and Hypertrophy Adaptations Between Low- vs. High-Load Resistance Training: A Systematic Review and Meta-analysis. J Strength Cond Res. 2017;31(12):3508–3523. 28834797 10.1519/JSC.0000000000002200

[28] Erskine RM, Jones DA, Williams AG, Stewart CE, Degens H. Inter-individual variability in the adaptation of human muscle specific tension to progressive resistance training. Eur J Appl Physiol. 2010;110(6):1117–1125. 20703498 10.1007/s00421-010-1601-9

[29] Stewart CE, Rittweger J. Adaptive processes in skeletal muscle: molecular regulators and genetic influences. J Musculoskelet Neuronal Interact. 2006;6(1):73–86. 16675891

[30] Charbonneau DE, Hanson ED, Ludlow AT, Delmonico MJ, Hurley BF, Roth SM. ACE genotype and the muscle hypertrophic and strength responses to strength training. Med Sci Sports Exerc. 2008;40(4):677–683. 18317377 10.1249/MSS.0b013e318161eab9 PMC2984550

[31] Willems SM, Wright DJ, Day FR, Trajanoska K, Joshi PK, Morris JA, Matteini AM, Garton FC, Grarup N, Oskolkov N, Thalamuthu A, Mangino M, Liu J, Demirkan A, Lek M, Xu L, Wang G, Oldmeadow C, Gaulton KJ, Lotta LA, Miyamoto-Mikami E, Rivas MA, White T, Loh PR, Aadahl M, Amin N, Attia JR, Austin K, Benyamin B, Brage S, Cheng YC, Cieszczyk P, Derave W, Eriksson KF, Eynon N, Linneberg A, Lucia A, Massidda M, Mitchell BD, Miyachi M, Murakami H, Padmanabhan S, Pandey A, Papadimitriou I, Rajpal DK, Sale C, Schnurr TM, Sessa F, Shrine N, Tobin MD, Varley I, Wain LV, Wray NR, Lindgren CM, MacArthur DG, Waterworth DM, McCarthy MI, Pedersen O, Khaw KT, Kiel DP, Consortium GA-ToF, Pitsiladis Y, Fuku N, Franks PW, North KN, van Duijn CM, Mather KA, Hansen T, Hansson O, Spector T, Murabito JM, Richards JB, Rivadeneira F, Langenberg C, Perry JRB, Wareham NJ, Scott RA. Large-scale GWAS identifies multiple loci for hand grip strength providing biological insights into muscular fitness. Nat Commun. 2017;8:16015. 29313844 10.1038/ncomms16015 PMC5510175

[32] Bouchard C, Sarzynski MA, Rice TK, Kraus WE, Church TS, Sung YJ, Rao DC, Rankinen T. Genomic predictors of the maximal O(2) uptake response to standardized exercise training programs. J Appl Physiol (1985). 2011;110(5):1160–1170. 21183627 10.1152/japplphysiol.00973.2010 PMC3098655

[33] Lonsdale J, Thomas J, Salvatore M, Phillips R, Lo E, Shad S, Hasz R, Walters G, Garcia F, Young N, Foster B, Moser M, Karasik E, Gillard B, Ramsey K, Sullivan S, Bridge J, Magazine H, Syron J, Fleming J, Siminoff L, Traino H, Mosavel M, Barker L, Jewell S, Rohrer D, Maxim D, Filkins D, Harbach P, Cortadillo E, Berghuis B, Turner L, Hudson E, Feenstra K, Sobin L, Robb J, Branton P, Korzeniewski G, Shive C, Tabor D, Qi L, Groch K, Nampally S, Buia S, Zimmerman A, Smith A, Burges R, Robinson K, Valentino K, Bradbury D, Cosentino M, Diaz-Mayoral N, Kennedy M, Engel T, Williams P, Erickson K, Ardlie K, Winckler W, Getz G, DeLuca D, MacArthur D, Kellis M, Thomson A, Young T, Gelfand E, Donovan M, Meng Y, Grant G, Mash D, Marcus Y, Basile M, Liu J, Zhu J, Tu Z, Cox NJ, Nicolae DL, Gamazon ER, Im HK, Konkashbaev A, Pritchard J, Stevens M, Flutre T, Wen X, Dermitzakis ET, Lappalainen T, Guigo R, Monlong J, Sammeth M, Koller D, Battle A, Mostafavi S, McCarthy M, Rivas M, Maller J, Rusyn I, Nobel A, Wright F, Shabalin A, Feolo M, Sharopova N, Sturcke A, Paschal J, Anderson JM, Wilder EL, Derr LK, Green ED, Struewing JP, Temple G, Volpi S, Boyer JT, Thomson EJ, Guyer MS, Ng C, Abdallah A, Colantuoni D, Insel TR, Koester SE, Little AR, Bender PK, Lehner T, Yao Y, Compton CC, Vaught JB, Sawyer S, Lockhart NC, Demchok J, Moore HF. The Genotype-Tissue Expression (GTEx) project. Nature Genetics. 2013;45(6):580–585. 23715323 10.1038/ng.2653 PMC4010069

[34] Cheret C, Willem M, Fricker FR, Wende H, Wulf-Goldenberg A, Tahirovic S, Nave KA, Saftig P, Haass C, Garratt AN, Bennett DL, Birchmeier C. Bace1 and Neuregulin-1 cooperate to control formation and maintenance of muscle spindles. EMBO J. 2013;32(14):2015–2028. 23792428 10.1038/emboj.2013.146 PMC3715864

[35] Morano M, Ronchi G, Nicolo V, Fornasari BE, Crosio A, Perroteau I, Geuna S, Gambarotta G, Raimondo S. Modulation of the Neuregulin 1/ErbB system after skeletal muscle denervation and reinnervation. Sci Rep. 2018;8(1):5047. 29568012 10.1038/s41598-018-23454-8 PMC5864756

[36] Balasubramanian A, Kawahara G, Gupta VA, Rozkalne A, Beauvais A, Kunkel LM, Gussoni E. Fam65b is important for formation of the HDAC6-dysferlin protein complex during myogenic cell differentiation. FASEB J. 2014;28(7):2955–2969. 24687993 10.1096/fj.13-246470 PMC4062822

[37] Brennan CM, Emerson CP, Jr., Owens J, Christoforou N. p38 MAPKs - roles in skeletal muscle physiology, disease mechanisms, and as potential therapeutic targets. JCI Insight. 2021;6(12): e149915. 34156029 10.1172/jci.insight.149915 PMC8262482

[38] Haun CT, Vann CG, Mobley CB, Osburn SC, Mumford PW, Roberson PA, Romero MA, Fox CD, Parry HA, Kavazis AN, Moon JR, Young KC, Roberts MD. Pre-training Skeletal Muscle Fiber Size and Predominant Fiber Type Best Predict Hypertrophic Responses to 6 Weeks of Resistance Training in Previously Trained Young Men. Front Physiol. 2019;10:297. 30971942 10.3389/fphys.2019.00297 PMC6445136

[39] Thomaes T, Thomis M, Onkelinx S, Fagard R, Matthijs G, Buys R, Schepers D, Cornelissen V, Vanhees L. A genetic predisposition score for muscular endophenotypes predicts the increase in aerobic power after training: the CAREGENE study. BMC Genet. 2011;12:84. 21967077 10.1186/1471-2156-12-84 PMC3193032

[40] Balshaw TG, Massey GJ, Maden-Wilkinson TM, Morales-Artacho AJ, McKeown A, Appleby CL, Folland JP. Changes in agonist neural drive, hypertrophy and pre-training strength all contribute to the individual strength gains after resistance training. Eur J Appl Physiol. 2017;117(4):631–640. 28239775 10.1007/s00421-017-3560-x

[41] Matta TT, Nascimento FX, Fernandes IA, Oliveira LF. Heterogeneity of rectus femoris muscle architectural adaptations after two different 14-week resistance training programmes. Clin Physiol Funct Imaging. 2015;35(3):210–215. 24750784 10.1111/cpf.12151

[42] Chung HC, Keiller DR, Roberts JD, Gordon DA. Do exercise-associated genes explain phenotypic variance in the three components of fitness? a systematic review & meta-analysis. PLoS One. 2021;16(10):e0249501. 34648504 10.1371/journal.pone.0249501 PMC8516263

[43] Wilk M, Zajac A, Tufano JJ. The Influence of Movement Tempo During Resistance Training on Muscular Strength and Hypertrophy Responses: A Review. Sports Med. 2021;51(8):1629–1650. 34043184 10.1007/s40279-021-01465-2 PMC8310485

